# Supplementary material for: A Pilot Interaction Analysis of Gut Microbiota and Peripheral Markers of Aging in Severe Psychiatric Disorders: A Role for Lachnoclostridium?
Source: Int J Mol Sci. 2023 Dec 18;24(24):17618. doi: 10.3390/ijms242417618 (PMC10744008; doi:10.3390/ijms242417618)
Supplement: Supplementary file 1 [file ijms-24-17618-s001.zip › ijms-2750384-supplementary.pdf]

Table S1. Comparison of dietary habits in the three samples (MDD, SCZ and HC)

Variable	Level	MDD (N=31)	SCZ (N=35)	HC (N=15)	p-value
Diet - N(%)	Mediterranean only	19 (61.3)	22 (62.9)	14 (93.3)	0.9
	Carbohydrates only	1 (3.2)	1 (2.9)	0 (0.0)	
	Vegetarian only	2 (6.5)	0 (0.0)	0 (0.0)	
	Mediterranean + high protein	3 (9.7)	3 (8.6)	0 (0.0)	
	Mediterranean + high protein + hypercaloric	1 (3.2)	0 (0.0)	0 (0.0)	
	Mediterranean + carbohydrates	1 (3.2)	2 (5.7)	1 (6.7)	
	Mediterranean + high protein + carbohydrates	1 (3.2)	2 (5.7)	0 (0.0)	
	Mediterranean + high protein + carbohydrates + hypercaloric	0 (0.0)	0 (0.0)	0 (0.0)	
	Mediterranean + hypercaloric + carbohydrates	1 (3.2)	1 (2.9)	0 (0.0)	
	Mediterranean + hypercaloric	0 (0.0)	0 (0.0)	0 (0.0)	
	High protein + carbohydrates	1 (3.2)	0 (0.0)	0 (0.0)	
	High protein + hypercaloric	0 (0.0)	0 (0.0)	0 (0.0)	
	Hypercaloric + carbohydrates	0 (0.0)	3 (8.6)	0 (0.0)	
	Hypercaloric + carbohydrates + vegetarian	1 (3.2)	0 (0.0)	0 (0.0)	
	Vegan only	0 (0.0)	1 (2.9)	0 (0.0)	

Abbreviations: HC = Healthy Controls; MDD = Major Depressive Disorder; N = number; NA = Not Available; SCZ = Schizophrenia.
